# A fully automatable enzymatic method for DNA extraction from plant tissues

**DOI:** 10.1186/1471-2229-5-23

**Published:** 2005-11-03

**Authors:** Jean-François Manen, Olga Sinitsyna, Lorène Aeschbach, Alexander V Markov, Arkady Sinitsyn

**Affiliations:** 1 University of Geneva, Conservatoire et Jardin Botaniques de la Ville de Genève, Impératrice 1, CH-1292 Chambésy/Genève, Switzerland; 2 Moscow State University, Chemistry Department, Vorobyevy Gory, 119899, Moscow, Russia

## Abstract

**Background:**

DNA extraction from plant tissues, unlike DNA isolation from mammalian tissues, remains difficult due to the presence of a rigid cell wall around the plant cells. Currently used methods inevitably require a laborious mechanical grinding step, necessary to disrupt the cell wall for the release of DNA.

**Results:**

Using a cocktail of different carbohydrases, a method was developed that enables a complete digestion of the plant cell walls and subsequent DNA release. Optimized conditions for the digestion reaction minimize DNA shearing and digestion, and maximize DNA release from the plant cell. The method gave good results in 125 of the 156 tested species.

**Conclusion:**

In combination with conventional DNA isolation techniques, the new enzymatic method allows to obtain high-yield, high-molecular weight DNA, which can be used for many applications, including genome characterization by AFLP, RAPD and SSR. Automation of the protocol (from leaf disks to DNA) is possible with existing workstations.

## Background

DNA extraction from plant tissues, unlike DNA isolation from mammalian tissues, remains difficult due to the presence of a rigid cell wall surrounding the plant cells. Currently used methods inevitably require a laborious mechanical grinding step, necessary to disrupt the cell wall for the release of DNA. The field of plant molecular biology is therefore at a disadvantage, especially when an automated high-throughput system for the isolation of PCR-ready genomic DNA is required in population genetics, species identification, biodiversity investigation, selection screening, food control and plant biotechnology. QIAGEN GmbH has developed a 96-well grinding method (MagAttract 96 Plant kit), but it requires a special mixer mill, a centrifugation step and consequently is not fully automatable.

Large scale automatable DNA mini-prep facilities were recently offered by several companies for animal tissues (e.g. DYNAL ASA, Oslo, Norway; AGOWA, Berlin, Germany; QIAGEN GmbH, Hilden, Germany; BILATEC AG, Mannheim, Germany; ROCHE Diagnostics, Rotkreuz, Switzerland; PROMEGA Corporation, Madison, WI, USA; SCIL Diagnostic GmbH, Martinsried, Germany). However, because of their cell wall, the automation of the isolation of DNA from plants needs improvements. Whereas animal tissues need only a lysis buffer containing detergents and proteinase K to release their DNA, plant tissues need in addition a mixture of carbohydrase enzymes able to digest the cell wall. Enzymatic digestion of the cell wall of leaf tissues is routinely used for the production of protoplasts but this approach was never adapted for routine isolation of DNA from plant tissue.

We describe here a new method for the lysis of plant tissues using a powerful cocktail of enzymes isolated from *Trichoderma longibrachiatum*, which digests the cell walls in order to liquefy the tissue without the need of grinding. The enzymatically released DNA is then isolated with commercially available magnetic beads.

## Results

**Figure 1:**
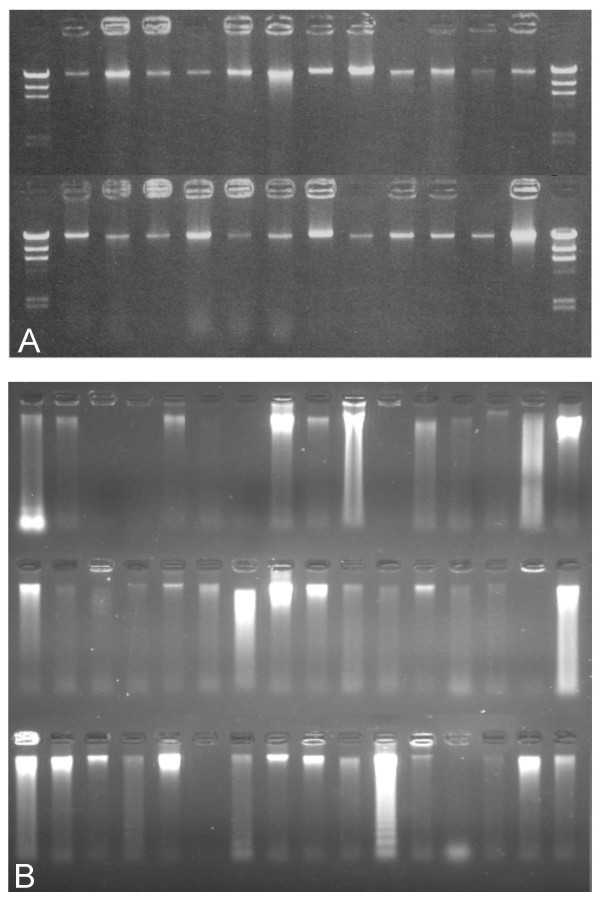
**Electrophoretic aspect of enzymatically isolated DNA. A: **Agarose gel electrophoresis of typical enzymatically isolated DNA from 24 different species (in the following order: *Phlomis fructicosa, Humulus lupulus, Veratrum album, Scilla bifolia, Astragalus gummifer, Vitis vinifera, Centaurea macrocephala, Narcissus pseudonarcissus, Allium ampeloprassum, Salvia officinalis, Viburnum carlesii, Colchicum speciosum, Triticum turgidum, Polygonum chinensis, Lathyrus vernus, Tilia sp., Caragana sophorifolia, Urtica dioica, Lilium henryi, Polygonum multiflorus, Geranium sp., Lupinus sp., Crocus albiflorus, Helleborus dumetorum*). After digestion, the DNA was isolated with Dynabeads^® ^DNA DIRECT™ Universal magnetic beads. One fourth of the isolated DNA was loaded. The first and last lines were loaded with 250 ng of lambda/HindIII DNA (500 ng, bottom half, right). **B: **Agarose gel electrophoresis of DNA of 42 randomly chosen species (in the following order: *Danae racemosa, Epimedium alpinum, Gladiolus palustris, Viburnum farreri, Euonymus bungeana, Weigela sp., Prunus padus, Rhodea japonica, Polygonatum multiflorum, Daphne japonica, Ribes petraeum, Asplenuim scolopendrium, Carex morrowii, Aruncus dioicus, Bletilla striata, Helleborus odoratus, Hedera helix, Brunnera macrophylla, Paeonia belladonna, Atropa belladonna, Solanum tuberosum, Beta vulgaris, Anethum graveolens, Allium fistulosum, Sison amomum, Uniola latifolia, Sinningia magnifica, Peperomia sp., Alnus sp., Tillia sp., Betula sp., Skimmia sp., Liriope spicata, Anthericum liliago, Inula ensifolia, Phlomis fruticosa, Lilium pumilum, Sorbaria sorbifolia, Dietes bicolor, Ilex aquifolium, Vitis vinifera, Setaria italica, Triticum aestivum, Nymphea sp., Pelargonium sp., Saintpaulia magungensis, Morinda sp., Zea mais*). After enzymatic digestion in half of a 96 microtitration plate, DNA was isolated using Wizard^® ^Magnetic 96 Plant System magnetic beads. One fourth of the isolated DNA was loaded.

Leaf disks from 24 different species were digested by 5 μl of the enzymatic cocktail in 50 μl of digestion buffer. Thirty μl of liquid containing cell debris were drawn up and released DNA was isolated using Dynabeads^® ^DNA DIRECT™ Universal kit (Dynal). Fig. 1A shows an agarose gel of 25% of the DNA isolated (10 μl). Most DNA are high-yield and of high-molecular weight. The amount of lambda DNA/Hind III loaded into the gel was 250 ng (or 500 ng, bottom half, right). The 23 kb band thus represented approximately 120 ng of DNA. The amount of plant genomic DNA obtained was variable from species to species. For some of them (*Humulus lupulus, Vitis vinifera, Narcissus pseudonarcissus, Tilia *sp., *Lilium henryi and Helleborus dumetorum*) the amount of loaded DNA was equal to or higher than 120 ng. As only 25% of the isolated DNA were loaded into the agarose gel, it can be estimated that the method permits the isolation of approximately 50 to 500 ng of genomic DNA from a leaf disk, depending on the species.

**Figure 2:**
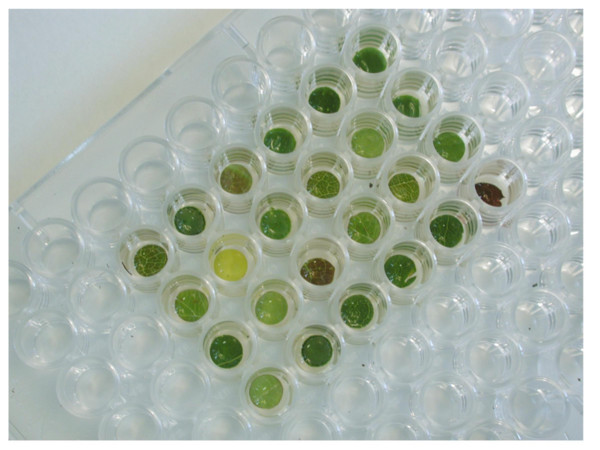
**Enzymatic disruption of leaf disks in a microtitration plate. **A flat bottom microtitration plate filled with 50 μl of digestion buffer and leaf disks of different species before the adding of the enzymatic cocktail.

In the experiment described above, species on which the method was previously tested were selected. In order to empirically examine to what extent the method works on different species, simultaneous extraction of 48 randomly chosen species was carried out in a microtitration plate (as shown on Fig. 2) using the Wizard^® ^Magnetic 96 Plant System kit (Promega) to isolate the released DNA (Fig. 1B). One fourth of the isolated DNA was loaded into the gel. In approximately 75% of the species, genomic DNA was visible. Several DNA extracts show partial degradation. No DNA is visible for *Gladiolus palustris, Viburnum farreri, Weigela sp, Prunus padus, Ribes petraeum, Betula sp, Sorbaria sorbifolia, Pelargonium sp, Saintpaulia magungensis*.

**Figure 3:**
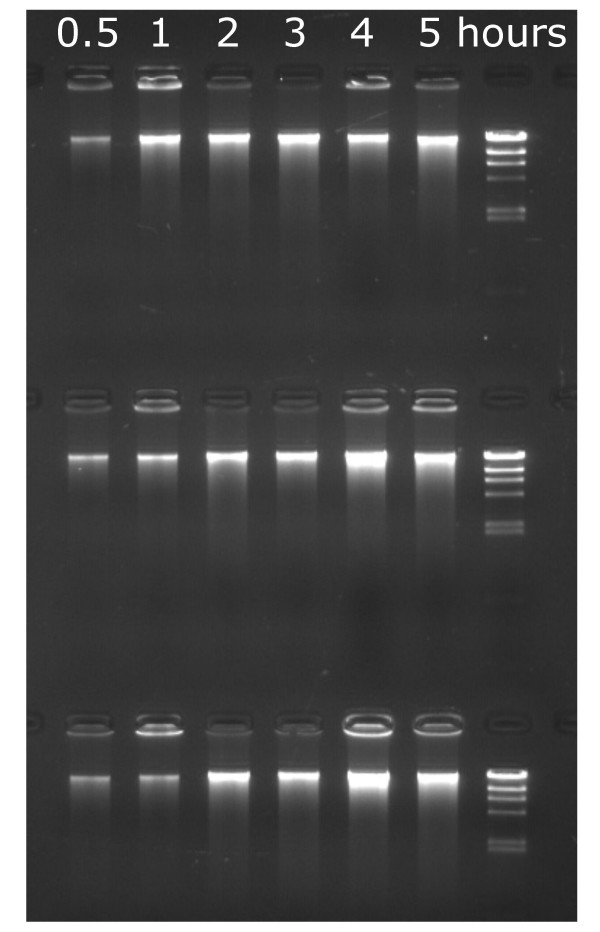
**Time scale DNA release from digesting leaf disks. **Triplicate essay of time scale DNA release from leaf disks of *Ilex aquifolium *at 0.5 to 5 hours of enzymatic digestion. Size marker: lambda/HindIII DNA.

In experiments described above, the cell wall digestion was done overnight for convenience. In order to follow the release of DNA at different times of enzymatic digestion, three 5 mm leaf disks from dry leaves of *Ilex aquifolium *were digested for 0.5 to 5 hours and the released DNA was isolated with the Wizard^® ^Magnetic 96 Plant System kit (Promega). Fig. 3 shows that some DNA is already released at 0.5 h and that 3 to 4 hours are sufficient to release most of the DNA from this species. A short digestion time (1 to 3 hours) is sufficient for soft leaves such as *Arabidopsis*, *Begonia*, *Brassica*, *Beta*, *Alium*, *Nicotiana*, *Triticum*, *Piper *...etc. However, incubation times need to be increased for *Fragaria*, *Ribes*, *Oryza*, *Soya*, *Zea *...etc. Thus, although 1 to 3 hours are generally enough, the incubation time for a given species is not foreseeable and the appropriate length of digestion has to be determined experimentally before undertaking large-scale DNA isolations.

**Figure 4:**
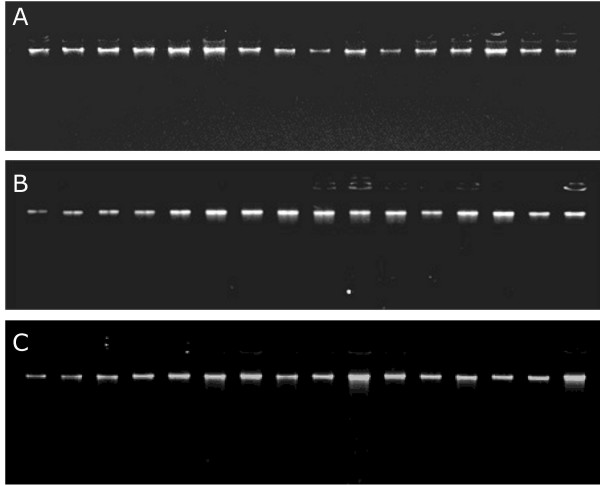
**Reproducibility of enzymatical isolation of DNA. **(A) From leaf disks of 16 individuals of *Aster amellus *(one tenth of the isolated DNA was loaded), (B) From 16 leaf disks of *Ilex aquifolium *(one fifth of the isolated DNA was loaded) and (C) From 16 seeds of *Allium porum *(one fifth of the isolated DNA was loaded) using Wizard^® ^Magnetic 96 Plant System magnetic beads.

**Figure 5:**
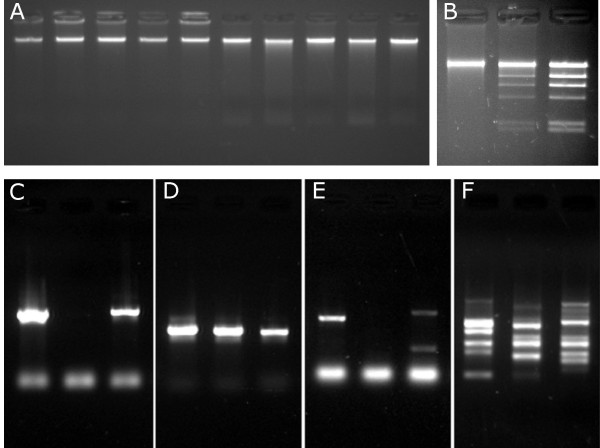
**Features and properties of enzymatically isolated DNA. A: **comparison of the amount of DNA isolated from *Ilex aquifolium *leaves by a conventional extraction method (lines 1–5) and by the enzymatic method described here (lines 6–10). In both case the amount of loaded DNA is one fifth of the DNA corresponding to one leaf disk. **B: **Study of the stability of lambda DNA/Hind III during the digestion of leaf disks of *Ilex*. Line 1: one leaf disk alone; line 2: one leaf disk and lambda DNA/Hind III; line 3: lambda DNA/Hind III alone. **C**, **D**, **E**, and **F**: PCR amplification of enzymatically isolated DNA from *Ilex aquifolium *(line 1), *Aster amellus *(line 2), and *Solanum tuberosum *(line 3). **C: **PCR amplification of the plastid *atpB-rbcL *spacer. **D: **PCR amplification of ITS/5.8S. **E: **PCR amplification of the nuclear encoded plastid glutamine synthetase. **F: **RAPD amplification.

The method is highly reproducible. Fig. 4 shows 16 DNA isolated from dry leaf disks of *Aster amellus *and *Ilex aquifolium *and from seeds of *Allium porum *(cut into 2 pieces, see Materials and Methods), using the Wizard^® ^Magnetic 96 Plant System kit (Promega). Fig. 5A shows a comparison of the amount of DNA isolated from *Ilex aquifolium *by a CTAB-based extraction method (lines 1–5, the protocol includes a treatment with RNase) and by the enzymatic method described here (lines 6–10) using the Wizard^® ^Magnetic 96 Plant System. In both cases the amount of loaded DNA is one fifth of the DNA corresponding to a leaf disk of approximately 3 mg (dry weight). The amount of isolated DNA is similar for both methods.

As indicated by Fig. 5B, lambda DNA/Hind III does not show degradation during incubation with the enzymatic mix, nor in the presence of an overnight digesting leaf disk of *Ilex *at 50°C. This indicates that the digestion mix does not contain active DNase in the condition used for digestion, and that in the case of *Ilex *endogenous plant DNase are inactivated by the digestion mix.

Fig. 5 also shows PCR amplification of a plastid sequence (Fig. 5C), a multi-copy nuclear sequence (Fig. 5D) and a single-copy nuclear sequence (Fig. 5E) from 1 μl of DNA isolated by the enzymatic method from *Ilex aquifolium, Aster amellus *and *Solanum tuberosum*. The primers have been designed for the genus *Ilex*. Thus the few negative PCR in other species probably result from primer mismatch and not from polymerase inhibition. RAPD amplifications are also shown (Fig. 5F).

**Figure 6:**
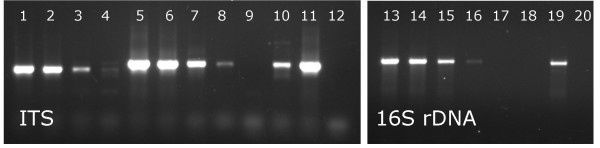
**Contamination checking: PCR markers for fungi and bacteria in DNA isolated by the enzymatic method. **Lines 1 to 12: Internal transcribed spacer (ITS) of ribosomal DNA amplified with eukaryotic specific universal primers ITS1 and ITS4 [17]. Lines 13 to 20: 16S ribosomal DNA amplified with prokaryotic specific universal primers 9f and 1429r [18]. Amplifications from respectively 10, 1, 0.1 and 0.01 pg of genomic DNA of *Trichoderma longibrachiatum *(lines 1 to 4), *Ilex aquifolium *(lines 5 to 8) and *Artrospira sp*. (lines 13 to 16). Amplifications of DNA isolated from a digestion mix alone (lines 9 and 17), a digestion mix "contaminated" with an *Ilex aquifolium *leaf disk removed after 10 min and further incubated overnight at 50°C (lines 10 and 18) and a mix digesting an *Ilex aquifolium *leaf disk overnight at 50°C (lines 11 and 19). Line 12 and 20: negative controls.

The enzymatic cocktail is produced from *Trichoderma longibrachiatum *fermentation and could be contaminated with its DNA. Moreover, in the case of a long overnight enzymatic digestion, there is a risk of contamination from bacteria or fungi covering the surface of plant tissues. To examine if such contamination could be a problem, fungi and bacteria specific PCR markers were tested on DNA extracted from (1) a digestion mix alone, or (2) a digestion mix "contaminated" with a *Ilex aquifolium *leaf disk removed after 10 min and further incubated overnight at 50°C, or (3) a mix digesting a *Ilex aquifolium *leaf disk overnight at 50°C (Fig. 6). No fungus (particularly *Trichoderma longibrachiatum*) or bacterial template could be detected. Instead, *Ilex aquifolium *templates are detected. Indeed, the ITS PCR product found in the digestion mix "contaminated" with a *Ilex aquifolium *leaf disk removed after 10 min and further incubated overnight at 50°C (line 10) has the same size as ITS of *Ilex *(larger than ITS of *Trichoderma*). Further sequencing demonstrated that this PCR product was an ITS sequence of *Ilex *and not of *Trichoderma *(data not shown). Similarly, the prokaryotic 16S rDNA sequence obtained from the mix digesting an *Ilex aquifolium *leaf disk overnight at 50°C (line 19) represented the plastid (prokaryotic) 16S rDNA of *Ilex *(data not shown), and not a bacterial sequence.

## Discussion

To the best of our knowledge, only a few non-grinding methods for isolation of DNA from plant tissue have been proposed, but only low amounts of DNA are generally obtained. Jhingan [1] followed by Williams and Ronald [2] proposed a chemical method using potassium ethyl xanthogenate that damages the cell wall, subsequently disrupts cells and releases the DNA. The method involves many steps and the amount of DNA released is generally ten times lower than traditional methods [1] and than our enzymatic method. A non-grinding method is proposed by SIGMA (Extract-N-Amp Plant PCR kit). It is not based on enzymatic digestion of the cell wall and the leaf tissue usually does not appear to be degraded after the treatment with the lysis buffer. The DNA extract is extremely crude, of low DNA content and often contains PCR inhibitors. Consequently, a 10-fold dilution of the extract is necessary to dilute inhibitors and the template concentration is at the limit of detection. Another method is based on the squashing of plant tissues on a nylon membrane [3] and subsequent elution of the little amount of DNA bound to the membrane for PCR amplification. An adaptation of this method is commercialized by WHATMAN (FTA^® ^gene card). In conclusion, the advantage of our enzymatic non-grinding method of DNA extraction compared with the above-described methods is that a large amount of high-quality DNA is isolated and that it is fully automatable.

In a paper on the comparative analysis of different DNA extraction protocols from plant tissues, Csaikl et al. [4] wrote that "the problem of DNA extraction is still an important issue in the field of plant molecular biology" and that "a chemical tissue disruption method as used in mammalian cells might be the method of choice". Plant DNA purification is time-consuming and laborious. It is considered as the "bottleneck" of basic and applied research [5]. Thus there is a need for a quick, easy and automated method of plant DNA isolation. The method that we present here exactly fits this expectation.

For a few species (approximately 25%, based on our results, see Additional file 1 and Fig. 2B) the method is not effective, but simple modifications of the protocol (particularly the digestion buffer) is expected to resolve the problem in the future. As the chemistry of plant tissues (contrary to animal tissues) is highly variable depending of species, it is not surprising that variable results are obtained. It was exactly the same situation with traditional DNA extraction methods where "recalcitrant" species needed further adaptations [6,7]. There are two situations in which the described protocol does not work (see Additional file 1). In the first case, the leaf disk of some species is not digested by the enzymatic cocktail. This is because some particular chemical compounds inhibit the enzymatic cocktail. *Quercus *represents such a case and high level of polyphenols (tannin) is suspected. Modifications of the digestion buffer by the addition of polyvinyl pyrrolidone (PVP [8]), or polyvinyl polypyrrolidone (PVPP [9]) or polyethylene glycol (PEG [10]) in order to neutralize polyphenolic compounds could greatly improve the method for "recalcitrant" species. In the second case, the leaf disk is perfectly digested but DNA is not released or, most probably, is highly degraded. This could be due to the release of endogenous recalcitrant nucleases or oxidative polyphenols during the digestion. In other cases (see *Betula sp*. in Additional file 1) different results can be obtained according the season of leaf harvesting, as it can be expected because of the modification of the chemical composition of the cell wall during the year [11]. To deal with species-dependent variability, it is obviously necessary to determine the optimal digestion conditions for each plant sample. In fact, the duration of incubation is not a problem because the protocol is entirely automatable from solid leaf disks to the PCR-ready DNA. Even if, in some case, it could be longer than mechanical grinding in reaction tube or plate, any human intervention is needed.

## Conclusion

In summary, the protocol is simple and reliable, does not require grinding, centrifuging, or the use of hazardous chemicals. A large number of samples can be processed simultaneously, and full automation of the protocol is possible with existing workstations. Many different species were successfully tested. The method can be adapted to each species by modification of the digestion buffer, of the amount of the enzymatic cocktail added during the digestion or of the digestion time.

The method is perfectly adapted to situations when high-throughput isolation of PCR-ready genomic DNA is required. Moreover, because of the high-yield and high-molecular weight DNA reliably obtained, sensitive PCR-based techniques could be applied: AFLP (Amplified Fragment Length Polymorphism), RAPD (Random Amplified Polymorphic DNA), SSR (Simple Sequence Repeat polymorphism).

## Methods

### Enzymatic cocktail

A mixture of cell wall degrading enzymes was isolated from *Trichoderma longibrachiatum *Rifai (strain TW-1, deposited in the Russian Collection of Microorganisms under the number VKMF-3934D). The fermenting medium (7 liters) consisted of wheat bran (25 g/L), solid corn steep (25 g/L), hydrolyzed starch (45 g/L), mineral salts and fed by lactose (25% solution at feeding rate of 50 ml/h) after the first 48 hours of fermentation. The fermentation was carried out at 32°C for 144 h. Extracellular secreted enzymes were then isolated by centrifugation (5000 g for 30 min.) and concentrated by ultrafiltration (molecular weight cut-off 10 kD) at 200–250 mg/ml of protein. The obtained enzymatic cocktail was used directly for DNA isolation from plant tissues. It contains, among others, cellulases, beta-glucanases, xylanases, mannanases, xyloglucanases, pectinases, glycosidases (such as beta-glucosidae, beta-xylosidase, alpha-L-arabinofuranosidase, alpha-galactosidase). Additional file 2 gives some enzymatic activities of the cocktail, as assayed according to Ghose [12]. Ribosomal DNA from *Trichoderma longibrachiatum *was not detected by PCR in the enzymatic cocktail, and cellulase from this organism is in the GRAS list (Generally Recognized As Safe) of the US Food and Drug Administration  under the number §184.1250.

The enzymatic cocktail remains stable at least for two years at 4°C. Substantial aliquots of the enzymatic preparation can be obtained from the first author.

### Plant tissues

One hundred and fifty six plant species from the Botanical Garden of Geneva were tested with the described enzymatic method of DNA isolation (Additional file 1). Leaf tissue was used in most cases and some seeds were also tested as indicated.

### Protocols

Leaf disks (5 mm in diameter) were incubated in 50 μl of digestion buffer (175 mM EDTA [pH 8.0], 100 mM sodium acetate [pH 4.6], 1% triton X100) and 5 μl of the enzymatic cocktail. After digestion (50°C with constant agitation from 3 to 16 hours, depending of species), 30 μl of liquid containing cell debris were drawn up and 200 μl of Dynabeads^® ^DNA DIRECT™ Universal (Dynal ASA, Oslo, Norway) was added. The protocol of DNA isolation was then conducted according the manufacturer's instructions in 1.5 ml microtubes.

Alternatively, leaf disks of 48 randomly chosen species were digested simultaneously overnight in the same conditions on a sealed flat bottom microtitration plate (as shown on Fig. 2). Genomic DNA was further isolated using the Wizard^® ^Magnetic 96 Plant System (Promega Corporation, Madison, WI, USA) and the MagnaBot^® ^96 Magnetic Separation Device, according to the manufacturer's instructions. For both protocols, DNA was eluted in 40 μl of TE8 (10 mM Tris-HCl, 1 mM EDTA, pH 8.0.). Fresh leaves were generally used, but silica gel-dried leaf tissue can also be digested. As well as leaf tissues, seed tissues were tested. To allow the enzyme solution to penetrate the seed tissue, seeds were broken into pieces of 1–3 mm in side, and one piece was used for DNA isolation. To examine the amount of DNA isolated, 10 μl of the eluted DNA was loaded on a 1% agarose gel containing ethidium bromide and the DNA band was compared with a known amount of lambda DNA /Hind III loaded into the gel.

### Stability of the DNA during the enzymatic digestion of plant tissue

One μg of lambda DNA/Hind III was added to the digestion mixture alone or in the presence of a leaf disk of *Ilex aquifolium *and incubated overnight at 50°C. DNA was then isolated with the Wizard^® ^Magnetic 96 Plant System (Promega) and one fifth of this DNA was loaded for agarose gel electrophoresis.

### Comparison with a conventional method of DNA extraction

The amount of DNA isolated by a method of DNA extraction based on CTAB (hexadecyltrimethylammonium bromide, [13]) was compared to the amount of DNA isolated by the enzymatic method described here for dry leaf tissue of *Ilex aquifolium*. Known amounts (from 14 to 53 mg) of liquid nitrogen-ground leaf tissue of *Ilex *were conventionally extracted and the isolated DNA was re-dissolved in the proportion of 50 μl of TE buffer per leaf disk (approximately 3 mg), the proportion used in the enzymatic method. The amounts of DNA were then compared by agarose gel electrophoresis.

### Genomic DNA analysis

PCR amplifications of a plastid fragment (the *atpB-rbcL *spacer, [14]), a multi-copy nuclear sequence (ribosomal ITS/5.8S, [15]) and a single-copy nuclear sequence (nuclear encoded plastid glutamine synthetase, [16]) were tested on DNA isolated from a leaf disk of *Ilex aquifolium*, *Aster amellus *and *Solanum tuberosum*. One μl of isolated DNA were used in 25 μl of standard PCR reactions (annealing temperature of 55°C). RAPD amplifications were tested with primer 5' CGGCCCCTGT using 1 μl of the isolated DNA was added to 25 μl of standard PCR reaction (annealing temperature of 37°C).

## Authors' contributions

JFM conceived of the method, carried out preliminary experiments and drafted the manuscript. OS and AVM checked and analyzed the biological and chemical activities of the enzymatic cocktail. LA accumulated and interpreted the data. AS designed the enzymatic cocktail.

## Supplementary Material

Additional File 1 List of species investigated. Click here for file

Additional File 2 Typical enzymatic activities and properties of the cocktail used for plant DNA isolation. Click here for file
