# Supplementary material for: A fully automatable enzymatic method for DNA extraction from plant tissues
Source: BMC Plant Biol. 2005 Nov 3;5:23. doi: 10.1186/1471-2229-5-23 (PMC1298311; doi:10.1186/1471-2229-5-23)
Supplement: Additional File 1 — List of species investigated. [file 1471-2229-5-23-S1.doc]

**List of species investigated.** Leaf disks were mostly tested (seed tissues when indicated). In bold economically important species.

**Tissue digested and genomic DNA isolated (125 species):**

*Acer campestre, Aesculus hippocastanum, Allium ampeloprassum, Allium fistulosum,* ***Allium porrum*** (seed)*, Alnus sp., Anethum graveolens, Anthericum liliago,* ***Arabidopsis thaliana****, Aristolochia macrophylla, Aruncus dioicus, Asparagus officinalis , Asplenium scolopendrium, Astragalus gummifer, Atropa belladonna, Begonia sp.,* ***Beta vulgaris****,* ***Betula sp*.** (harvested in May), *Bletilla striata, Bombax sp.,* ***Brassica oleracea,*** *Brunnera macrophylla, Buxus sempervirens,* ***Camellia sinensis****, Caprinus sp., Caragana sophorifolia, Cardamine heptaphylla, Carex morrowii, Centaurea macrocephala, Cercidiphylum japonicum, Chamaedorea microspadix, Clematis sp.,* ***Coffea arabica,*** *Colchicum speciosum, Crocus albiflorus, Cyclamen purpurascens, Cymbidium pendulum, Danae recemosa, Daphne japonica, Dendrobium moschatum, Dietes bicolor, Dipteracanthus devosianus, Epimedium alpinum, Eranthis hyemalis, Eryngium planum, Euonymus bungeana, Euphorbia leuconeura, Euphorbia rigida,* ***Fragaria sp.****, Fumaria capreolata, Gladiolus palustris , Geranium sp., Gloxinia sp.,* ***Glycine max*** (seed*),* ***Gossypium sp****., Hedera helix, Helleborus dumetorum, Helleborus odoratus, Hibiscus magnifica, Humulus lupulus, Hyacintus orientalis, Hypoestes sp., Ilex aquifolium, Impatiens sodenii, Inula ensifolia,* ***Lactuca sativa***(seed)*, Lathyrus vernus, Lilium henryi, Lilium pumilum, Liriope spicata, Lonicera caerulea****, Lupinus sp.****,* ***Lycopersicon esculentum****,* ***Mentha piperita****, Morinda sp., Narcissus pseudonarcissus,* ***Nicotiana tabacum****, Nymphea sp., Oreopanax sp., Oryza sativa, Paeonia belladonna, Paeonia suffruticosa, Palisota mannii,* ***Papaya sp****., Peperomia sp., Petasites albus, Phlomis fruticosa,* ***Piper sp****., Polygonatum multiflorum, Polygonum chinensis, Polygonum multiflorus, Potentilla aurea, Primula pubescens, Primula acaulis, Psychotria guadeloupensis, Rheum palmatum, Ribes petraeum, Rhodea japonica , Saintpaulia magungensis,* ***Salvia officinalis****, Saponaria officinale, Scilla bifolia, Setaria italica, Sinningia sp., Sinningia magnifica, Sison amomum, Skimmia sp.,* ***Solanum tuberosum****, Sorbus aria, Stachytarpheta sp., Tilia sp., Trichantha filifera,* ***Triticum aestivum***(leaf and seed)*, Triticum spelta , Triticum turgidum,* ***Tulipa sp.,*** *Uniola latifolia*, *Urtica dioica, Vanhoutea sp., Veratrum album, Viburnum carlesii,* ***Vitis vinifera****, Weigelia floribunda, Weigelia precox,* ***Zea mais***(leaf and seed),

**Leaves digested, but no genomic DNA visible on agarose gels (20 species):** *Asplenium sp., Betula sp.* (harvested in July), *Crassula sp., Epiphyllum oxypetatum, Eranthemum sp., Eriobotrya japonica, Miltonia spectabilis, Myrmecodia sp., Parthenocissus inserta, Pelargonium sp, Prunus padus, Ribes alpinum, Ribes petraeum, Saintpaulia magungensis, Schomburgkia undulata, Sorbaria sorbifolia, Taxus sp, Uniola latifolia, Viburnum farreri, Weigela sp.*

## Leaves non digested (11 species): *Betula papyrifera, Bixa orellana, Corylus avellana, Liquidambar formosana, Magnolia grandifolia, Medinilla magnifica, Miscanthus sinensis, Pinus wallichina, Prunus laurocerasus, Quercus sp., Syzygium jambos.*
