# Supplementary material for: A fully automatable enzymatic method for DNA extraction from plant tissues
Source: BMC Plant Biol. 2005 Nov 3;5:23. doi: 10.1186/1471-2229-5-23 (PMC1298311; doi:10.1186/1471-2229-5-23)
Supplement: Additional File 2 — Typical enzymatic activities and properties of the cocktail used for plant DNA isolation. [file 1471-2229-5-23-S2.doc]

**Typical enzymatic activities and properties of the cocktail used for plant DNA isolation.** The activities are measured as the release of reducing sugars assayed by the Nelson-Somogy test [19].

| **Enzyme** | **MW**  **(kD)** | **pI** | **Type of**  **Action** | **Activities ^(1)^** | | | | | **pH_opt_** | **T_opt_**  **(°C)** | **Stability ^(2)^ (50^0^C)** | |
| --- | --- | --- | --- | --- | --- | --- | --- | --- | --- | --- | --- | --- |
|  |  |  |  | **CMC-ase^(3)^** | **β-Glucan-ase** | **Xylan-ase** | **Avicelase** | **Other Substrates** |  |  | **pH 5** | **pH 7** |
| **EG I** | 57 | 4.6 | endo | 24 | 44 | 8 | 0.22 | 45 (XG) ^(4)^ | 4.5-5.0 | 60 | 100% | 1h, 60% |
| **EG II** | 48 | 5.5 | endo | 15 | 27 | 0 | 0.18 |  | 4.7 | 70-75 | 100% | 0.7h, 0% |
| **EG III** | 25 | 7.5 | endo | 11 | 19 | 0.6 | 0.06 | 9 (XG) ^(4)^ | 6.0 | 60 | 87% | 1h, 20% |
| **CBH I** | 65 | 3.7 | exo | 0.3 | 0.2 | 0 | 0.2 |  | 4.0-4.5 | 60 | 100% | 1h, 42% |
| **CBH II** | 53 | 6.3 | exo | 1.7 | 2.5 | 0 | 0.19 |  | 4.5-5.0 | 70-75 | 100% | 0.7h, 0% |
| **XYL I** | 21 | 5.5 | endo | 0 | 0 | 8 | 0 |  | 2.0-2.5 | 50 | 96% | 0.3h, 0% |
| **α-L-Ara** | 55 | 7.4 | exo | 0 | 0 | 1.2 | 0 | 14 (*p*NPH-α-L-Ara) ^(5)^ | 3.5-4.0 | 60 | 97% | 0.2h, 0% |
| **exo-β-1,3-Glu** | 74 | 8.9 | exo | 0.4 | 16 | 0 | 0 | 30 (Laminarin) | 5.0 | 70 | 80% | 1h, 40% |
| **PGA** | 45 | 6.0 | endo | 0 | 0 | 0 | 0 | 200 (PGU) ^(6)^ | 5.0 | 60 | 85% | 0.2h, 12% |
| **XG** | 85 | 4.2 | exo | 2.6 | 3.5 | 0 | 0 | 44 (XG) ^(4)^ | 5.3 | n. d. | 83% | n. d. |

EG I, II, III – endo-glucanases I, II, III

CBH I, II – cellobiohydrolases I, II

Xyl I – xylanase I

α-L-Ara – α-L-Arabinofuranosidase

exo-β-1,3-Glu – exo-β-1,3-Glucanase

PGA – polygalacturonase (pectinase)

XG – xyloglucanase

(1) – Specific activity (Units/1 mg of purified enzyme)

(2) – Residual activity after incubation during 3 hours, except when indicated.

(3) – Activity toward soluble cellulose

(4) – Xyloglucan (Tamarind)

(5) – p-nitrophenil-α-L-Arabinofuranozide

(6) – Polygalacturonic acid
